# The Effectiveness of Pap and Visual Inspection With Acetic Acid (VIAA) Tests in Cervical Dysplasia Screenings During the COVID-19 Pandemic

**DOI:** 10.7759/cureus.27364

**Published:** 2022-07-27

**Authors:** Moraima Lagos-Castillo, María Guevara-Vizcarra, Felipe Paredes-Campos, Sathyatej Kosuri, Gustavo Vilchez

**Affiliations:** 1 Medical Technology, Universidad Nacional Federico Villareal, Lima, PER; 2 Obstetrics and Gynecology, Universidad Nacional Federico Villareal, Lima, PER; 3 Ophthalmology, Universidad Nacional Federico Villareal, Lima, PER; 4 Obstetrics and Gynecology, University of Missouri - Kansas City, Kansas City, USA

**Keywords:** sensitivity and specificity, colposcopy, acetic acid, early detection of cancer, cytological techniques, vaginal smears, uterine cervical neoplasms, papanicolaou test, sars-cov-2, covid-19

## Abstract

Objective

This study was aimed at analyzing the validity and reliability of the Papanicolaou (Pap) test and visual inspection with acetic acid (VIAA) tests for cervical dysplasia screenings during the COVID-19 pandemic.

Material and methods

This was a retrospective study of patients 21 years or older seen at the Luis Negreiros Primary Care Center in Lima, Peru between 2020 and 2021, who underwent cervical dysplasia screening (Pap or VIAA). Relevant information regarding patient age, date of service, and Pap and VIAA results were collected. Parallel form reliability was analyzed with chi-square tests, and phi, contingency and Cramer's V coefficients. The validity of these tests was analyzed through the calculation of the sensitivity, specificity, and positive and negative predictive values with confidence intervals. A p-value less than 0.05 indicated statistical significance.

Results

From 4,503 records, the sensitivity, specificity, and positive and negative predictive values for Pap were 0.87 (0.81-0.92), 1.0 (1.0-1.0), 1.0 (1.0-1.0) and 0.99 (0.98-0.99), respectively, and those for VIAA were 0.22 (0.14-0.31), 0.10 (0.10-0.10), 0.53 (0.38-0.69) and 0.10 (0.10-0.10), respectively. Test validity varied slightly according to patient age and the year of testing. The correlation, although significant, was inverse; chi-square = 39.18, p <0.001, phi = -0.60, contingency = 0.51 and Cramer’s V = -0.59.

Conclusion

The validity and reliability of Pap testing and VIAA for cervical dysplasia screening significantly decreased during the COVID-19 pandemic. The correlation between these tests, although significant, was inverse. More larger-scale studies are needed to confirm these findings and understand the reasons underlying the decreased effectiveness of these tests.

## Introduction

The COVID-19 pandemic has significantly affected many health care systems worldwide [1]. Primary health care is a major part of the health care system that has been substantially impaired by this pandemic [2]. The number of patients presenting to outpatient clinics for routine oncologic screening tests, such as cervical dysplasia screening tests, has significantly decreased [3].

The Papanicolaou (Pap) test and the visual inspection test with acetic acid (VIAA) are screening tests routinely used to rule out malignant or premalignant lesions of the cervix. During the COVID-19 pandemic, several studies have indicated that the number of patients presenting for outpatient screening tests decreased significantly because of several factors, including fears of being exposed to sick individuals and acquiring COVID-19. However, whether the demographics of the groups of patients who were able to present for testing have changed significantly has not been studied.

Because COVID-19 is more common in patients with oncologic diseases [4], and viral infections may increase the risk of malignancy [5], studying the reasons underlying the decrease in screening tests performed is essential, including how any changes in demographics might potentially have affected the sensitivity and specificity of these screening tests. Because the validity and reliability of screening tests, such as Pap and VIAA, depend on factors such as the prevalence of the disease, possible changes in the testing frequency, and the demographics of patients who undergo these tests might have significantly affected test accuracy and predictive ability.

Because of the high morbidity and mortality of cervical cancer [6], analysis of the effectiveness of Pap and VIAA tests is crucial to alert healthcare workers regarding potential changes in the efficacy of these tests, as well as to inform policymakers of the potential need to adjust current health care policies to detect cervical cancer more efficiently. Therefore, the objective of the present research study was to analyze the validity and reliability of Pap and VIAA as screening tests for the detection of premalignant and malignant diseases of the cervix during the COVID-19 pandemic.

## Materials and methods

The present study used a non-experimental, cross-sectional, retrospective design. Data were collected for patients who attended the Luis Negreiros Health Care Center in Lima, Peru, between 2020 and 2021 (during the COVID-19 pandemic). The inclusion criteria were patients over the age of 21 who underwent cervical dysplasia screening with a Pap or VIAA test. Both tests were performed following the same institutional protocols throughout the study period by the same group of certified nurse midwives trained specifically to perform these tests. The Pap test was performed in the routine fashion by placing the speculum in the vagina for visualization of the cervix and using an Aylesbury spatula to circumferentially scrape the outer opening of the external os and collect a sample of cervical cells. Subsequently, an endocervical brush was used to collect samples from the endocervix by rotating the brush 180 degrees. The VIAA test is a recognized direct visualization technique that, different from colposcopy, does not require the use of magnification, and was performed by placing the speculum in the vagina for direct visualization and examination of the cervix, with subsequent application of acetic acid with the assistance of cotton. After one minute, the cervix was inspected again to note any lesions or colour changes. No biopsy was considered part of the VIAA test. Both tests were interpreted by pathology physicians.

Information relevant to the study, such as patient age, year of visit, and the results of Pap and VIAA tests and definitive diagnosis (as documented in the chart and based on subsequent diagnostic testing such as colposcopy, biopsy, endocervical curettage, or diagnostic excisional procedures) were obtained retrospectively from the medical records and collected in a Microsoft Excel 365 file for subsequent analysis.

All statistical analyses were performed in Statistical Analytical Software (SAS) package version 9.4. The normality of numerical variables was calculated with graphical methods such as Q-Q plots, and numerical methods such as deviation, kurtosis, and the Shapiro-Wilk test. Numerical variables with a non-parametric distribution are represented as medians and interquartile ranges. Categorical variables are expressed as the number of cases and percentages and were compared with chi-square tests. To study the parallel reliability of the Pap and VIAA tests, chi-square tests were used, and phi, contingency, and Cramer's V coefficients were calculated. 

To study the validity of these screening tests, we calculated diagnostic testing accuracy parameters, such as sensitivity, specificity, positive predictive value, and negative predictive value, with their respective standard errors and 95% confidence intervals. To study the effects of demographic variables, such as patient age and year of the screening visit, on the validity of the Pap and VIAA tests, we calculated the same accuracy testing parameters in subsets according to each of these categories. To analyze the effects of disease prevalence on the validity of the diagnostic tests, we plotted the positive and negative predictive values according to the estimated prevalence calculated from the sensitivity and specificity obtained for each screening test. For all analyses, a p-value less than 0.05 was considered statistically significant. For all coefficients, a value greater than 0.8 was considered to indicate optimal correlation.

The protocol of the present research study Nro 192-CIEI-OlyD-GRPS-ESSALUD-2021 was exempted from full review by the Institutional Review Board of the Federico Villareal National University.

## Results

After the application of the inclusion criteria, a total of 4,503 patient records were included in the study. Of these, 4,503 (100.0%) underwent a Pap test, and 110 (2.4%) underwent a VIAA test. The patients’ median age (and interquartile range) was 43 (34-52) years. A total of 2,749 patients (61.0%) were tested in 2020, and 1,754 (38.9%) were tested in 2021.

The correlation between the Pap and VIAA test results was statistically significant: chi-square = 39.18, p-value < 0.001, phi = -0.60, contingency = 0.51, Cramer's V = -0.59. The validity parameters of both tests are shown in Table 1. The Pap test had a sensitivity (and 95% CI) of 0.87 (0.81-0.92), specificity of 1.00 (1.00-1.00), positive predictive value of 1.00 (1.00-1.00) and negative predictive value of 0.99 (0.98-0.99). The VIAA test had a sensitivity of 0.22 (0.14-0.31), specificity of 0.10 (0.10-0.10), positive predictive value of 0.53 (0.38-0.69) and negative predictive value of 0.10 (0.10-0.10).

**Table 1:** Accuracy Levels of Diagnostic Tests (N=4503)

	Estimate	Standard Error	Confidence Interval (95%)
Pap test (Prevalence = 2.7%)			
Sensitivity	0.87	0.02	0.81 – 0.92
Specificity	1.00	0.00	1.00 – 1.00
Positive Predictive Value	1.00	0.00	1.00 – 1.00
Negative Predictive Value	0.99	0.01	0.98 – 0.99
Visual inspection with acetic acid (Prevalence = 19.1%)			
Sensitivity	0.22	0.04	0.14 - 0.31
Specificity	0.10	0.10	0.10 – 0.10
Positive Predictive Value	0.53	0.07	0.38 – 0.69
Negative Predictive Value	0.10	0.10	0.10 – 0.10

Test validity parameters according to the year of study are shown in Table 2. For the Pap test, a slight decrease in sensitivity was detected from 2020 to 2021: sensitivity (and 95% CI) = 0.91 (0.84-0.98) vs. 0.83 (0.75-0.91). For the VIAA test, a slight decrease was also detected from 2020 to 2021: sensitivity 0.26 (0.13-0.40) vs 0.19 (0.08-0.30); positive predictive value 0.68 (0.46-0.91) vs. 0.43 (0.23-0.63). The same test validity parameters according to patient age are shown in Table 3. The Pap test showed a slight increase in sensitivity with age in the 19-39 year age group compared with the group over 64 years of age, sensitivity (95% CI) = 0.80 (0.71-0.89), 0.93 (0.88-0.99) and 1.00 (1.00-1.00), respectively. In the VIAA test group, a slight decrease in sensitivity was seen in the 40-64 year group compared with the 19-39 year group: 0.24 (0.12-0.35) vs. 0.21 (0.08-0.34); moreover, a slight increase in the positive predictive value was observed: 0.48 (0.29-0.66) vs. 0.66 (0.39-0.93).

**Table 2:** Diagnostic Testing Accuracy Parameters According to Testing Year (N=4503)

Diagnostic Testing Accuracy Parameters According to Testing Year				
Year			2020	2021
Pap test				
			Estimate (95% confidence interval)	
Sensitivity			0.91 (0.84 – 0.98)	0.83 (0.75 – 0.91)
Specificity			1.00 (1.00 – 1.00)	1.00 (1.00 – 1.00)
Positive Predictive Value			1.00 (1.00 – 1.00)	1.00 (1.00 – 1.00)
Negative Predictive Value			0.99 (0.98 – 0.99)	0.99 (0.98 – 0.99)
Visual inspection with acetic acid				
			Estimate (95% confidence interval)	
Sensitivity			0.26 (0.13 - 0.40)	0.19 (0.08 - 0.30)
Specificity			0.10 (0.10 – 0.10)	0,10 (0.10 – 0.10)
Positive Predictive Value			0.68 (0.46 - 0.91)	0.43 (0.23 - 0.63)
Negative Predictive Value			0.10 (0.10 - 0.10)	0,00 (0.00 – 0.00)

**Table 3:** Diagnostic Testing Accuracy Parameters According to Patient Age (N= 4503)

Diagnostic Testing Accuracy Parameters According to Patient Age			
Maternal Age	21 - 39 years	40 - 64 years	> 64 years
Pap test			
	Estimate (95% confidence Interval)		
Sensitivity	0.80 (0.71 – 0.89)	0.93 (0.88 – 0.99)	1.00 (1.00 – 1.00)
Specificity	1.00 (1.00 – 1.00)	1.00 (1.00 – 1.00)	1.00 (1.00 – 1.00)
Positive Predictive Value	1.00 (1.00 – 1.00)	1.00 (1.00 – 1.00)	1.00 (1.00 – 1.00)
Negative Predictive Value	0.99 (0.98 – 0.99)	0.99 (0.98 – 0.99)	1.00 (1.00 – 1.00)
Visual Inspection with Acetic Acid			
	Estimate (95% confidence Interval)		
Sensitivity	0.24 (0.12 - 0.35)	0.21 (0.08 – 0.34)	N/A
Specificity	0.10 (0.10 – 0.10)	0.10 (0.10 – 0.10)	
Positive Predictive Value	0.48 (0.29 – 0.66)	0.66 (0.39 – 0.93)	
Negative Predictive Value	0.10 (0.10 – 0.10)	0.10 (0.10 – 0.10)	

**Figure 1:**
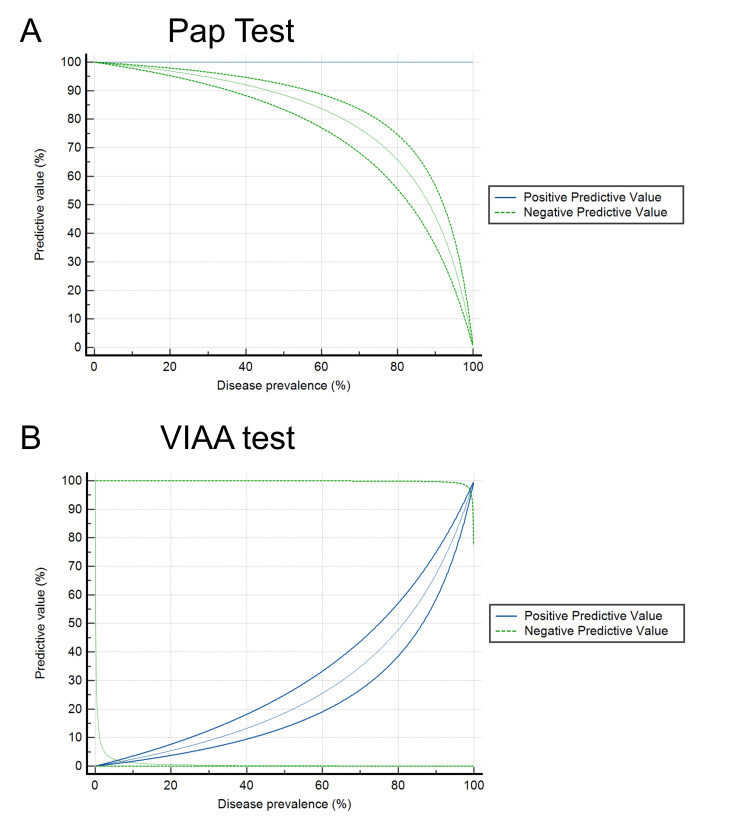
Predictive Values of Pap test and Visual Inspection With Acetic Acid (N = 4,503). Predictive values (positive and negative) according to the estimated prevalence according to sensitivity and specificity for screening tests for cervical cancer of Pap test (A) and visual inspection with acetic acid (B). In A, it is observed that the negative predictive value of the Pap test maintains a gradual decrease to an approximate prevalence of 80.0%. In B, it is observed that the positive predictive value gradually increases to an estimated prevalence of approximately 70.0%.

The predictive values (positive and negative) and their confidence intervals according to the prevalence estimated for both tests on the basis of the obtained sensitivity and specificity are shown in Figure 1. For the Pap test, the negative predictive value gradually decreased to an estimated prevalence of approximately 80.0%. For the VIAA test, the positive predictive value gradually increased to an estimated prevalence of approximately 70.0%.

## Discussion

The key findings of the current study are that during the COVID-19 pandemic, screening tests for cervical dysplasia, specifically the Pap and VIAA tests, have been negatively affected. Many recent studies have demonstrated the negative effects of the COVID-19 pandemic on screening tests for cervical dysplasia [7]. These effects has been sufficiently severe that some consider the screening programs for this disease to be in a state of crisis [8-10]. Other studies have also shown an increasing number of women diagnosed with premalignant lesions [11-13]. Although many reasons may explain these negative effects, the decreased number of patients undergoing screening may be part of the problem [10,14-16]. 

Other social factors negatively affecting the performance of these tests include patients’ fear of exposing themselves to sick individuals when attending their screening appointments and consequently acquiring COVID-19 [17]. Similarly, owing to fear of illness, the number of health care providers reporting to work has also markedly decreased [18,19]. Furthermore, several studies have shown substantial delays in obtaining the results of these tests [11]. Because of this state of crisis, many countries worldwide have been called to take urgent action in studying how to improve the quality of screening tests [20]. 

Many studies have examined the accuracy of the Pap and VIAA tests for the detection of cervical dysplasia before the COVID-19 pandemic. In 2019, Vahedpoor et al. reported a sensitivity and specificity of 29.7% and 85.5%, respectively, for the Pap test [21]. In recent years, Egede and colleagues have reported a sensitivity of 80.0% and a specificity of 91.8% [22]. In the same year, Huy et al. reported a sensitivity and specificity of 58.0% and 85.2%, respectively [23]. One of the most recent systematic reviews and meta-analyses on the subject has reported a sensitivity and specificity of 57% and 95%, respectively, for the Pap test [24]. 

Several studies also analyzed the precision of the VIAA test for the detection of cervical dysplasia before the COVID-19 pandemic. In 2019, Vahedpoor and collaborators reported a sensitivity and specificity of 94.6% and 81.6%, respectively [21]. Rosado and collaborators reported a sensitivity and specificity of 99% and 5%, respectively [25]. In 2018, Egede and colleagues reported a sensitivity of 73.3% and a specificity of 96.5% [22]. In that same year, Huy and collaborators reported a sensitivity and specificity of 88.8% and 43.8%, respectively [23]. One of the most recent systematic reviews and meta-analyses on the subject has reported a sensitivity and specificity of 79% and 84%, respectively, for the VIAA test [24]. However, comparable data during the COVID-19 pandemic are sparse. 

The correlation between the Pap and VIAA test results for cervical dysplasia screening was also studied before the COVID-19 pandemic. In 2012, Consul et al. reported a statistically significant correlation between the results of these two tests [26]. In the same year, Arun and colleagues also reported a significant correlation, with a kappa value of 0.5 [27]. However, data regarding the correlation between, or effectiveness of, cervical dysplasia screening tests during the COVID-19 pandemic are scarce. Although many studies have analyzed the decreased number of screening tests and the underlying reasons, information regarding accuracy testing parameters (sensitivity, specificity, and positive and negative predictive values) for Pap and VIAA tests is limited. Therefore, the present study is one of the first of its kind to analyze not only the precision values but also the correlation between the results of these tests. 

The findings of the present study have many clinical implications. The results could be used to alert healthcare workers regarding the negative effects of COVID-19 on these tests and the need for greater caution in applying and interpreting these results. The importance of external evaluation of cervical cytology and computer system implementation has been addressed previously [28,29]. In addition, revision of current health care policies appears warranted to reverse the substantial damage that this pandemic has inflicted on health care programs, specifically those for the prevention of cervical cancer [30].

The results of the present study also open the door to more research opportunities in this field. Continued study of the effects of the COVID-19 pandemic on these and several other screening tests is crucial. Although malignancy is well known to increase the risk of severe COVID-19 [4], whether SARS-CoV-2 infection increases the risk of malignancy has not been well studied [5]. Qualitative studies are also required to investigate the need for health care policy changes to reverse the damage caused by cervical malignancies and to improve detection rates.

The present study has several strengths. To our knowledge, in contrast to the many other studies focusing on studying the frequency of screening and the factors affecting it, this study is one of the first to examine the validity and reliability of the Pap and VIAA tests during the COVID-19 pandemic. The sample size was very homogeneous and sufficient to obtain significant results with data from recent years.

This study is not exempt from limitations. Because of the research objective and study design, no detailed follow-up information on the specific diagnostic tests performed was obtained for further analysis. Likewise, because of the need to use a retrospective study design to achieve an adequate sample, the study was based on data collected through a database and review of medical records wherein missing data and potential coding errors might have decreased the quality of the data.

## Conclusions

In conclusion, the validity and reliability of Pap and VIAA tests for cervical uterine dysplasia screening have significantly decreased during the COVID-19 pandemic. The correlation between the results of these tests, although significant, was found to be inverse. The results of this study may be used to alert healthcare workers regarding the negative effects of COVID-19 on these tests and to alert policymakers regarding the need to revise current health care policies. These findings also open the door to further research opportunities in the field to confirm these findings and understand the reasons for the decreased efficacy of these tests, while improving patient outcomes.
